# Metabolomic Phenotype of Hepatic Steatosis and Fibrosis in Mexican Children Living with Obesity

**DOI:** 10.3390/medicina59101785

**Published:** 2023-10-07

**Authors:** Nayely Garibay-Nieto, Karen Pedraza-Escudero, Isabel Omaña-Guzmán, María José Garcés-Hernández, Eréndira Villanueva-Ortega, Mariana Flores-Torres, José Luis Pérez-Hernández, Mireya León-Hernández, Estibalitz Laresgoiti-Servitje, Berenice Palacios-González, Juan Carlos López-Alvarenga, Mauricio Lisker-Melman, Felipe Vadillo-Ortega

**Affiliations:** 1 Pediatric Obesity Clinic and Wellness Unit, General Hospital of Mexico, Mexico City 06720, Mexico; gngaribay@hotmail.com (N.G.-N.); karenpedrazaescudero@outlook.com (K.P.-E.); nutricion.isa@gmail.com (I.O.-G.); majogarces7@gmail.com (M.J.G.-H.); villorte@yahoo.com (E.V.-O.); 2 Unidad de Vinculación de la Facultad de Medicina, UNAM, Instituto Nacional de Medicina Genómica, Mexico City 14610, Mexico; mflores@inmegen.edu.mx; 3 Hepatology Clinic, Gastroenterology Department, General Hospital of Mexico, Mexico City 06720, Mexico; josluiperez@hotmail.com; 4 Research Unit, General Hospital of Mexico, Mexico City 06720, Mexico; leonhmireya@gmail.com; 5 Clinical Health Sciences, School of Medicine and Health Sciences, Tecnológico de Monterrey, Monterrey 64849, Mexico; estibalitz.laresgoiti@itesm.mx; 6 Laboratorio de Envejecimiento Saludable, Centro de Investigación Sobre el Envejecimiento, Instituto Nacional de Medicina Genómica, Mexico City 14610, Mexico; bpalacios@inmegen.gob.mx; 7 Department of Population Health & Biostatistics, University of Texas Rio Grande Valley, Edinburg, TX 78539, USA; juan.lopezalvarenga@utrgv.edu; 8 Division of Gastroenterology, Washington University School of Medicine, St. Louis, MO 63110, USA; mlisker@wustl.edu

**Keywords:** MASLD, metabolomics, children, obesity, amino acids, acylcarnitine

## Abstract

*Background and Objectives*: Metabolic-dysfunction-associated steatotic liver disease or MASLD is the main cause of chronic liver diseases in children, and it is estimated to affect 35% of children living with obesity. This study aimed to identify metabolic phenotypes associated with two advanced stages of MASLD (hepatic steatosis and hepatic steatosis plus fibrosis) in Mexican children with obesity. *Materials and Methods*: This is a cross-sectional analysis derived from a randomized clinical trial conducted in children and adolescents with obesity aged 8 to 16 years. Anthropometric and biochemical data were measured, and targeted metabolomic analyses were carried out using mass spectrometry. Liver steatosis and fibrosis were estimated using transient elastography (Fibroscan^®^ Echosens, Paris, France). Three groups were studied: a non-MASLD group, an MASLD group, and a group for MASLD + fibrosis. A partial least squares discriminant analysis (PLS-DA) was performed to identify the discrimination between the study groups and to visualize the differences between their heatmaps; also, Variable Importance Projection (VIP) plots were graphed. A VIP score of >1.5 was considered to establish the importance of metabolites and biochemical parameters that characterized each group. Logistic regression models were constructed considering VIP scores of >1.5, and the receiver operating characteristic (ROC) curves were estimated to evaluate different combinations of variables. *Results*: The metabolic MASLD phenotype was associated with increased concentrations of ALT and decreased arginine, glycine, and acylcarnitine (AC) AC5:1, while MASLD + fibrosis, an advanced stage of MASLD, was associated with a phenotype characterized by increased concentrations of ALT, proline, and alanine and a decreased Matsuda Index. *Conclusions*: The metabolic MASLD phenotype changes as this metabolic dysfunction progresses. Understanding metabolic disturbances in MASLD would allow for early identification and the development of intervention strategies focused on limiting the progression of liver damage in children and adolescents.

## 1. Introduction

In the last decade, an increase in the prevalence and incidence of obesity has been observed globally, which has led to an increase in comorbidities associated with this complex disease. Metabolic-dysfunction-associated steatotic liver disease (MASLD), previously known as non-alcoholic fatty liver disease (NAFLD) [1], is the most common cause of chronic liver disease in children and adolescents, with an estimated prevalence of about 35% in those with obesity [2]. The prevalence varies according to the studied populations and the diagnostic methods used, which remain imprecise and sometimes inaccessible in daily practice [3]. The spectrum of abnormalities ranges from simple intrahepatic fat accumulation in more than 5% of the parenchyma, followed by steatohepatitis (SH) characterized by inflammation and the necrosis of hepatocytes with various degrees of fibrosis. These abnormalities may eventually progress to cirrhosis and hepatocellular carcinoma [4]. There is increasing evidence that suggests that the pathophysiology of the disease is complex and involves multiple genetic, epigenetic, and environmental factors. Cross-sectional and longitudinal studies have found an association between MASLD and the degree of adiposity, which is a strong predictor of the development and progression of this metabolic dysfunction [2]. On the other hand, Schwimmer et al. reported a histological prevalence of SH in 54% of children with obesity and fibrosis in 11.8% [5], which denotes the presence of liver damage from the early stages of life.

MASLD requires an intentional search in children with obesity, particularly in children with manifestations of metabolic syndrome given the high rate of progression to severe forms suggested in this age group [6]. Even though liver biopsy remains the gold standard for MASLD diagnosis, its invasiveness, likelihood of sampling error, and poor feasibility as a follow-up method are primary and significant limitations. Some biochemical indexes have particularly emerged as a need to discover non-invasive tools that could accurately diagnose hepatic fibrosis, which has been recognized as a strong predictor of hepatic cirrhosis [7]. The AST/ALT ratio, AST/platelet ratio index (APRI), FIB-4 score, NAFLD fibrosis score [8], and plasma Cytokeratin-18 [9] have been validated in the pediatric population using the gold standard liver biopsies but showed limited performance and emphasized the prevailing urgency of having more precise diagnostic methods [10,11].

Conventional ultrasound (B-mode US) is a frequently used method, with a sensitivity of 79.9% and a specificity of 86.2%, for the diagnosis of moderate to severe steatosis, with an area under the receiver operating characteristic curve (ROC area) of 0.87 [12,13]; however, it has limitations for the detection of SH and fibrosis in addition to the drawback of being operator-dependent. Other methods, such as magnetic resonance spectroscopy and magnetic resonance proton density fat fraction (MRS-PDFF), have shown higher accuracy for differentiating degrees of steatosis, comparing moderate–severe vs. mild degrees with an ROC area of 0.87 (95% CI [0.80–0.94]), and an ROC area of 0.79 (95% CI [0.70, 0.87]) for distinguishing severe steatosis from mild and moderate forms [14]. However, they cannot detect fibrosis, and their ability to screen and diagnose is limited; moreover, they are unfortunately not always available. Draijer et al. published a systematic review that evaluated several diagnostic methods that analyzed the presence of liver fibrosis in the pediatric population. Transient elastography using FibroScan^®^ showed high accuracy with an ROC area of 0.98 (95% CI [0.90–0.99]), resulting in a sensitivity and a specificity > 90% at a threshold of 5.1 kPa [15]. This method has shown superiority over traditional biochemical scores [16].

On the other hand, based on the pathophysiologic mechanisms behind MASLD and SH, some studies have attempted to identify useful biomarkers to predict and act as signatures of MASLD and its progression stages [17]. To achieve this purpose, metabolomic approaches (targeted and non-targeted) have been used. However, most studies have been carried out with respect to the adult population, and only a few have been carried out with respect to children and adolescents [17,18,19,20].

Moreover, none of these studies were conducted in resident populations in Latin America, where the prevalence of childhood overweight and obesity and associated comorbidities is one of the highest worldwide [21].

Targeted metabolomic analysis has evaluated amino acid and lipid alterations. In children residents in Los Angeles, CA, USA, it was observed that MAFLD was associated with metabolites related to the metabolism of fatty acid, glutathione, and tryptophan [17]. Otherwise, in children with severe obesity from Vienna [19] and children with obesity from USA [18], it was found that those with MASLD have higher concentrations of branched-chain amino acids (BCAAs) and short-chain acylcarnitines when compared with children without this condition. In addition, models that included these metabolites have been shown to have an adequate capacity to identify MASLD in children [18,19] independently of other metabolic alterations such as insulin resistance and obesity.

Using an untargeted approach, consistent with the above, in children and adolescents self-identified as Hispanic with a BMI of ≥85th, it was observed that those with MASLD have alterations in some amino acid pathways (BCAAs, tyrosine, methionine, and cysteine metabolism) and lipid metabolism (lipogenesis and fatty acid metabolism) [20].

The objective of this study is to identify metabolic phenotypes associated with two progression stages of MASLD (hepatic steatosis and hepatic steatosis plus fibrosis) in children with obesity.

## 2. Materials and Methods

### 2.1. Study Design and Subjects

We conducted a cross-sectional comparative study that was a branch of a randomized clinical trial where the main objective was to evaluate several physical activity interventions in pediatric patients with obesity who were included in a multidisciplinary lifestyle change intervention program.

Children and adolescents aged 8 to 16 years who had not been previously treated and were sent to the Pediatric Obesity Clinic at the Child Welfare Unit in the General Hospital of Mexico were eligible. Obesity was defined using the Centers for Disease Control and Prevention criteria (body mass index (BMI ≥ 95th percentile according to age and sex)), and only Class 1 obesity participants were included (BMI < 120% over 95th percentile according to age and sex) [22]. Exclusion criteria included Class 2 or 3 obesity, genetic or endocrine obesity, systemic illness, familial dyslipidemia, alcohol consumption, and drug-induced liver injury. A history of liver diseases was ruled out.

The study was approved by the Hospital’s Institutional Research, Ethics, and Biosafety for Human Research Committees (Number DI/17/311/03/028) and registered in ClinicalTrials.gov (NCT03552367). Parents and children provided written informed consent and assent, respectively. This trial was conducted in accordance with the 1975 and 2013 Declarations of Helsinki and adhered to the Good Clinical Practice Guidelines issued by the International Conference of Harmonization. All patient data were protected in compliance with the Health Insurance Portability and Accountability Act (HIPAA).

### 2.2. Measurements

#### 2.2.1. Anthropometry

The measurements were performed by trained pediatricians and nutritionists after a standardization procedure. Total body weight was measured relative to participants dressed in light clothes, and a mechanical column scale was used (to the nearest 0.1 kg); standing height was measured using a standard stadiometer board mounted to the wall (to the nearest 0.1 cm). Waist circumference was measured at the midway point between the last costal cartilage and the anterosuperior iliac crest with a non-stretchable fiberglass measuring tape at the end of a breath. Body composition was determined using Body Composition Analyzer Model IOI 353 (to 0.1 kg) (Jawon Medical Co, Gyeonggi-do, Republic of Korea) after a 12 h fasting period. We registered blood pressure using a digital sphygmomanometer, following a fifteen-minute rest and using an appropriate cuff size for the children and adolescents’ upper arms. Pubertal assessments according to mammary, genital, and pubic statuses for both boys and girls and based on Marshall and Tanner staging were explored and defined by a pediatric endocrinologist [23]. BMI was calculated according to Quetelet’s Equation.

#### 2.2.2. Biochemical Evaluation

After 12 h of fasting, a venous blood sample was obtained to measure alanine aminotransferase (ALT), aspartate aminotransferase (AST), gamma-glutamyl transpeptidase (GGT), total cholesterol (TC), high-density lipoprotein cholesterol (HDL-Chol), low-density lipoprotein cholesterol (LDL-Chol), and triglycerides (Tg) using available commercial kits. To calculate Matsuda ISI, we performed an oral glucose tolerance test using 1.75 g/kg of the body weight of anhydrous glucose (to a maximum of 75 g). Samples were taken at 0, 30, 60, 90, and 120 min, and glucose and insulin were determined. The Matsuda-ISI (Insulin Sensitivity Index) was calculated as follows: 10,000/√ (fasting glucose [mg/dL] × fasting insulin [mU/L]) × (mean glucose × mean insulin) [24]. The HOMA-IR was calculated as follows: (fasting insulin [mU/L] × fasting glucose [mg/dL])/405 [25]. The glucose level was analyzed enzymatically with the use of commercially available reagents. Insulin was measured using Bio-Plex Pro Human diabetes insulin immunoassay by Bio-Rad (Hercules, CA, USA).

Tg, TC, and HDL-Chol were measured using standardized enzymatic methods, and LDL-Chol was calculated using Friedewald’s method.

#### 2.2.3. Evaluation of Liver Steatosis and Fibrosis

Liver steatosis and fibrosis were estimated using transient elastography (Fibros can^®^Echosens, Paris, France). The controlled attenuation parameter (CAP) was measured to detect liver fat accumulation, and the “M” 3.5 MHz probe (diameter 7 mm) or the “XL” 2.5 MHz probe (diameter 10 mm) was used if very thick adipose tissue was present. Participants were placed in a supine position, and the examiner situated the probe in the skin between the ribs, which vertically faced the right lobe of the liver. The cut-off point for predicting steatosis was CAP ≥ 225 dB/m [26]. Liver stiffness was measured in kPa. Fibrosis was diagnosed using the cut-off point proposed by Nobili and colleagues as ≥5.1 kPa [27]. The reported CAP (dB/m) and kPa data were the median of 10 measurements obtained by a trained investigator; only results with an interquartile range below 30% were valid. MASDL was defined as having abnormal CAP and/or kPa values. All measurements were performed by the same investigator.

Three groups were categorized according to hepatic abnormalities evaluated using Fibroscan: non-MASLD (children without steatosis and fibrosis), MASLD (children with steatosis and without fibrosis), and MASLD + fibrosis (children with steatosis and fibrosis).

#### 2.2.4. Targeted Metabolomic Determinations

A targeted metabolome analysis was carried out, including glucose, free carnitine, acylcarnitines (AC2, AC3, AC4, AC5, AC5:1, AC6, AC8, AC8:1, AC10, AC10:1, AC10:2, AC12, AC12:1, AC14, AC14:1, AC14:2, AC14OH, AC16, AC16:1, AC16:1OH, AC16OH, AC18, AC18:1, AC18:1OH, AC18:2, and AC18OH), arginosuccinate (ASA), and 12 L-amino acids (alanine, glycine, arginine, methionine, proline, arginine, valine, leucine, phenylalanine, tyrosine, citrulline, and ornithine). The above was achieved by using a Quattro Micro API (MicroMass, Cary, NC, USA) tandem mass spectrometer (MS-MS). All procedures for sample preparation and MS-MS analysis were performed using a NeoBase Non-derivatized kit (PerkinElmer, Waltham, MA, USA) according to the manufacturer’s protocol. The serum was dried in filter papers, and single disks were punched from each spot using a 3 mm punch. One disk was used per well. Using a multichannel pipette, 190 μL of extraction solution containing a mixture of the respective stable isotope-labeled internal standards was added to each well. The plate was covered with aluminum foil, shaken at 650× *g*, and incubated for 30 min at 30 °C. The plate was finally placed in the auto-sampler for analysis. All results were expressed in μM.

### 2.3. Statistical Analysis

To describe the studied population, the mean and standard deviations (SD) were estimated for continuous variables, and proportions and frequencies were calculated for categorical variables. To evaluate the differences between groups (non-MASLD, MASLD, and MASLD + fibrosis), ANOVA and exact Fisher tests were performed, and Bonferroni post hoc tests were conducted to identify the differences between groups.

Metabolite concentrations and biochemical data were median-normalized, square- or cube-root-transformed, and range-scaled. A partial least squares discriminant analysis (PLS-DA) was performed to identify the discrimination between study groups (non-MASLD vs. MASLD, non-MASLD vs. MASLD + fibrosis, and MASLD vs. MASLD + fibrosis). Variable importance projection (VIP) plots were graphed to visualize the metabolites that contributed the most to the discrimination of samples, and heatmaps based on the VIP scores were made to visualize the differences between the groups’ metabolite concentrations and biochemical data.

Finally, logistic regression models were constructed considering the metabolites and biochemical data with VIP scores >1.5 as independent variables (different combinations of these variables were tested), and the MASLD groups (Non-MASLD vs. MASLD and Non-MASLD vs. MASLD + fibrosis) as dependent variables. To evaluate whether different sets of metabolites and biochemical data can identify MASLD and MASLD + fibrosis groups, receiver operating characteristic (ROC) curves were estimated. To define the metabolomic phenotype, we selected models with a reduced number of metabolites and an adequate ROC area.

A *p*-value of <0.05 was considered significant. The analyses were performed using MetaboAnalyst 5.0 and Stata version 15.

## 3. Results

A total of 79 children and adolescents were included in the study; of them, 52.7% were female with a mean age of 11.74 ± 2.52 years. For males, the mean age was 10.66 ± 1.71. Of the total sample, 39% were classified as prepubertal, 32.4% belonged to the early puberty group, and 28.4% belonged to advanced puberty. There were no significant differences in the Tanner stage between the study groups (non-MASLD, MASLD, and MASLD + fibrosis). No significant differences in sex distribution were found in the prepubertal group; nonetheless, significantly higher proportions of males within early puberty and females within advanced puberty were evident (*p* = 0.001). No differences regarding the duration of obesity were found among pubertal or sex categories. Even though the total sample corresponded to Class 1 obesity, the BMI-z score was significantly higher in males (*p* = 0.033). The sex distribution was not different among groups. The overall frequency of MASLD (only hepatic steatosis) was 59.5%, and for MASLD + fibrosis, it was 21.6%. Anthropometric, biochemical, and metabolic characteristics are shown in Table 1. Abdominal adiposity, insulin resistance, and ALT increased as liver abnormalities progressed.

### 3.1. Metabolic Phenotypes According to the Progression of MASLD

#### 3.1.1. Non-MASLD and MASLD

**Figure 1:**
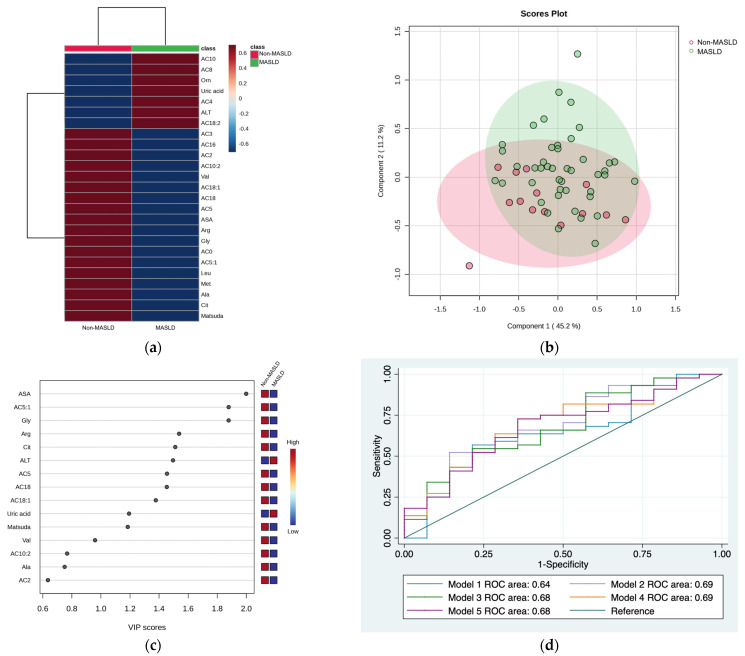
Serum metabolite and biochemical profile in children with MASLD. (**a**) Hierarchical heatmap for MASLD diagnosis, and the red and blue colors represent increased and decreased concentrations, respectively. (**b**) The PLS-DA plot shows the separation between groups (accuracy 0.69; R2 0.22; Q2 −0.20). (**c**) The VIP analysis represents the relative contribution of metabolites to the variance among groups (a high VIP score indicates a greater contribution of metabolites to the separation of the groups), and the red and blue boxes on the right indicate whether the metabolite concentration is increased (red) or decreased (blue). (**d**) Comparison of the ROC areas (Model 1: ASA + AC5:1 + glycine + arginine + citrulline; Model 2: Model 1 + ALT; Model 3: AC5:1 + glycine + arginine + citrulline + ALT; Model 4: AC5:1 + glycine + arginine + ALT; Model 5: glycine + arginine + ALT).

The hierarchical heatmap (Figure 1a) shows the increased or decreased variables in children from the MASLD and non-MASLD groups. Children with MASLD had higher concentrations of AC10, AC8, AC4, AC18:2, ornithine, uric acid, and ALT than children without MASLD. PLS-DA discriminated two clusters in the non-MASLD and MASLD groups (Figure 1b). VIP analyses identified ASA, AC5:1, glycine, arginine, and citrulline as the variables discriminated between the groups (VIP score of >1.5) (Figure 1c). We evaluated five combinations of metabolites and enzymes with VIP scores higher than 1.5 (Model 1: ASA + AC5:1+ glycine + arginine + citrulline; Model 2: Model 1 + ALT; Model 3: AC5:1 + glycine + arginine + citrulline + ALT; Model 4: AC5:1 + glycine + arginine + ALT; Model 5: glycine + arginine + ALT). Table 2 shows the different models tested and their respective ROC areas. No significant differences between ROC areas were observed; therefore, the selected model was Model 4 because this model included fewer variables than the others and an acceptable ROC area (ROC area = 0.69) (Figure 1d). Model 2 had a similar ROC curve of 0.69 but included six variables. The coefficients for Model 2 were as follows: Pr(y = 1|x) = 1.31 − 1.043 AC5:1 − 2.093 Gly − 1.427 Arg + 4.096 ALT.

#### 3.1.2. Non-MASLD and MASLD + Fibrosis

**Figure 2:**
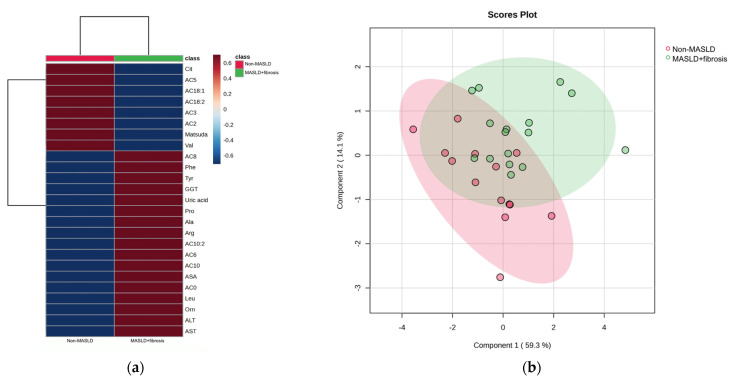

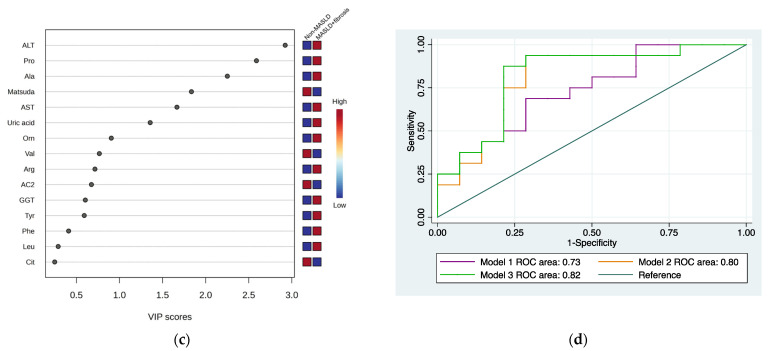
Serum metabolite and biochemical profile in children with MASLD + fibrosis. (**a**) Hierarchical heatmap for MASLD + fibrosis diagnosis, and the red and blue colors represent increased and decreased concentrations, respectively. (**b**) The PLS-DA plot shows the separation between groups (accuracy 0.74; R2 0.48; Q2 0.20). (**c**) The VIP analysis represents the relative contribution of metabolites to the variance among groups (a high VIP score indicates a greater contribution of the metabolites to the separation of the groups), and the red and blue boxes on the right indicate whether the metabolite concentration is increased (red) or decreased (blue). (**d**) Comparison of the ROC areas (Model 1: ALT + proline + alanine; Model 2: ALT + proline + alanine + Matsuda Index; Model 3: ALT + proline + alanine + Matsuda Index + AST).

The hierarchical heatmap (Figure 2a) shows that children with MASLD + fibrosis had increased concentrations of phenylalanine, tyrosine, GGT, uric acid, proline, alanine, arginine, leucine, ornithine, carnitine, medium-chain acylcarnitine (AC8, AC10:2, AC6, and AC10), carnitine, ASA, ALT, and AST than children without MASLD. The MASLD and non-MASLD groups were separated into two clusters in the PLS-DA (Figure 2b). The metabolites and biochemical parameters that discriminated better between the study groups (VIP score > 1.5) were ALT, proline, alanine, Matsuda Index, and AST (Figure 2c). Based on the VIP scores, we tested three combinations of metabolites and biochemical data to select an MASLD phenotype (Model 1: ALT + proline + alanine; Model 2: ALT + proline + alanine + Matsuda Index; Model 3: ALT + proline + alanine + Matsuda Index + AST) (Figure 2d and Table 2). We further considered the ROC areas and a reduced number of metabolites while maintaining an adequate capacity to classify individuals. The inclusion of AST in Model 3 did not significantly improve the ROC curve; therefore, Model 2 was selected: Pr(y = 1|x) = 0.283 + 0.625 ALT + 1.07 Proline − 1.98 Ala − 2.98 Matsuda Index (ROC area = 0.80).

#### 3.1.3. MASLD and MASLD + Fibrosis

**Figure 3:**
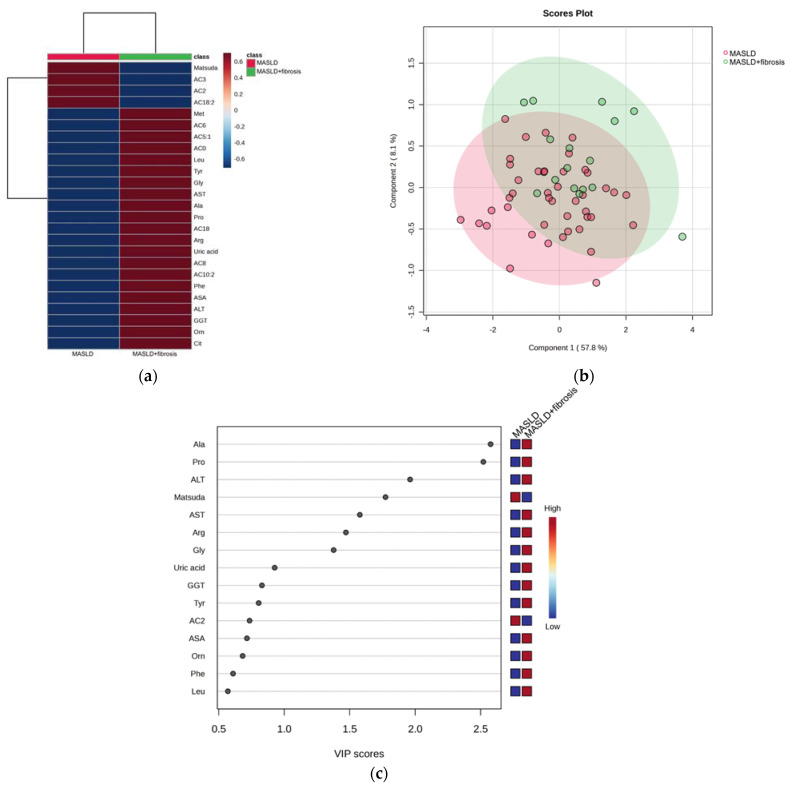
Serum metabolite and biochemical profile in children with MASLD. (**a**) Hierarchical heatmap for MASLD + fibrosis diagnosis, and the red and blue colors represent increased and decreased concentrations, respectively; (**b**) the PLS-DA plot shows the separation between groups (accuracy 0.68; R2 0.29; Q2 0.01); (**c**) the VIP analysis represents the relative contribution of metabolites to the variance among groups (a high VIP score indicates a greater contribution of the metabolites to the group separation), and the red and blue boxes on the right indicate whether the metabolite concentration is increased (red) or decreased (blue).

Once the phenotypes for MASLD and MASLD + fibrosis were established, we aimed to identify the differences between MASLD and MASLD + fibrosis. The heatmap showed that children with MASLD + fibrosis had higher concentrations of methionine, leucine, glycine, alanine, proline, arginine, phenylalanine, ornithine, citrulline, carnitine, AC5:1, medium-chain acylcarnitine (AC6, AC8, and AC10:2), AC18, ALT, AST, and GGT than children with only MASLD (Figure 3a).

The PLS-DA discriminated into two clusters (Figure 3b). According to the VIP analysis, the metabolites and biochemical parameters that discriminate the samples (VIP score > 1.5) were alanine, proline, ALT, Matsuda Index, and AST (Figure 3c).

## 4. Discussion

In the present study, we described the metabolomic phenotypes associated with two stages of MASLD progression (MASLD (hepatic steatosis) and MASLD + fibrosis (hepatic steatosis + fibrosis)) in Mexican children with obesity compared to those with obesity but without MASLD. According to logistic regression models, MASLD was associated with a phenotype characterized by increased concentrations of ALT and decreased arginine, glycine, and AC5:1 (tiglylcarnitine). On the other hand, MASLD + fibrosis, a progression stage of MASLD, was associated with a phenotype characterized by increased concentrations of ALT, proline, and alanine and a decreased Matsuda Index. This metabolic signature also identified MASLD + fibrosis children from the MASLD group. A major metabolic change in children affected with MAFLD + fibrosis results in the increased availability of amino acids in circulation.

The VIP analyses identified other metabolite differences between groups; however, we wanted to select a reduced set of metabolites and biochemical data capable of identifying MASLD and MASLD + fibrosis groups that might be useful in clinical practice.

ALT is a marker of hepatic damage and is widely used to screen MASLD, SH, and hepatic fibrosis in adults and children [28,29,30,31]. However, there are cases of children with MASLD who show normal levels of ALT [30], which makes evident the sensitivity limits of ALT for screening MASLD. In the present study, ALT was a consistent biomarker that discriminated between the non-MASLD group and the two groups representing stages in MASLD progression (MASLD and MASLD + fibrosis). Moreover, we observed that ALT discriminated the group of MASLD + fibrosis from MASLD, which means that this enzyme increased as MASLD progressed, and liver damage advanced. Therefore, we selected ALT as a part of the metabolic phenotype of MASLD and MASLD + fibrosis.

Although ALT elevation is part of MASLD and MASLD + fibrosis phenotypes, other metabolites acted as metabolic signatures in each group. The MASLD phenotype included decreased concentrations of arginine, glycine, and AC5:1. The ROC area of this model was below 0.70, and the VIP scores were lower than 2.0; therefore, the measurement of these metabolites was not enough to identify the MASLD phenotype. Despite the above, other studies have found alterations in some of these metabolites. Arginine is essential in the urea cycle and is a precursor of nitric oxide (NO) [32], creatine, and polyamines [33]. Furthermore, this amino acid regulates inflammation processes via the glutathione system’s activation, which enables pyruvate carboxylase upregulation and a significant decrease in reactive oxygen species production. The arginine NO synthase (NOS) pathway improves insulin secretion and sensitivity. Consequently, alterations in arginine metabolism are associated with endothelial damage and insulin resistance [34]. There is no evidence of arginine metabolism dysregulation in MASLD, but the decreased concentrations in these children could be a sign of glucose and insulin alterations that are known to be correlated with hepatic steatosis and obesity [35]. On the other hand, glycine participates in glutathione synthesis and the regulation of gene expression, among other functions [36]. Low concentrations of this amino acid have been related to cardiometabolic alterations in adults and children [37,38]. Consistent with our results, lower glycine concentrations have been observed in overweight children with MASLD than in children with overweight but without MASLD [39]. Moreover, in an untargeted metabolomic analysis performed on Hispanic adolescents with MASLD who are residents in the USA, a dysregulation in glycine metabolism was proposed [20]. AC5:1 is a short-chain acylcarnitine derived from isoleucine metabolism [40]. Lower concentrations of this acylcarnitine have been associated with inflammatory bowel diseases [41] and increased concentrations with respect to metabolic syndromes [42] in adults. Nevertheless, no studies have characterized AC5:1 concentrations in children with MASLD; therefore, more studies are needed to explain this finding.

The MASLD + fibrosis phenotype included, in addition to increased ALT, increased proline and alanine and a decreased Matsuda Index. Proline is essential in the structure and function of proteins and is a major component of collagen [43,44]. Hepatic fibrosis is the excessive accumulation of proteins in the extracellular matrix (ECM), mainly collagen, and it is a consequence of chronic liver injury and inflammation [45] associated with MASLD evolution [46]. As collagen synthesis requires high amounts of proline, this amino acid and hydroxyproline have been suggested as biomarkers of liver fibrosis [47]. Alanine is an amino acid with multiple biological functions, such as glucose synthesis, urea synthesis, and heme synthesis, among others [38]. Studies in children with MASLD have not found differences in alanine concentrations compared with controls. However, studies in the adult population have reported increased alanine in MAFDL [39] and MASLD + fibrosis patients [40]. Elevated ALT is coincident with increased serum concentrations of alanine, proline, and other amino acids in the MASLD + fibrosis group, supporting the metabolic funneling of amino acids for use in gluconeogenesis, which may explain the reduced insulin sensitivity in this group. The insulin resistance present in MASLD [25] is associated with an increase in the gluconeogenic rate [41], and it has been suggested that constant excessive gluconeogenesis participates in the progression from hepatic steatosis to fibrosis [41]; moreover, decreased Matsuda Index values in the MASLD + fibrosis phenotype indicate that these children had altered insulin sensitivity.

Increased ALT, proline, and alanine and a decreased Matsuda Index were characteristics of children with MASLD + fibrosis, which discriminated this group from the non-MASLD and MASLD subjects, indicating that these variables reflect specific changes that could be a consequence of the progression of MASLD to fibrosis. Consistent with our findings, a study conducted in a population of Mexican adults with obesity [48] found that a set of amino acids, including alanine and proline concentrations, predicted the Matsuda Index and MASLD, suggesting that these amino acids play a role in the development of insulin resistance and MASLD. According to our results, it seems that this metabolic signature characterized by higher alanine and proline concentrations and lower Matsuda Index is present since infancy. Most metabolomic studies conducted on children with MASLD have not evaluated the presence of fibrosis and, according to our results, it is important to separate hepatic steatosis from steatosis + fibrosis. Regarding this, with an untargeted approach, Kordy et al. [8] observed that children with SH and fibrosis had a different metabolomic signature than those with simple steatosis characterized by alterations in one-carbon metabolism, increased oxidative stress, and mitochondrial dysfunction.

From a practical and clinical point of view, it seems imperative to generate diagnostic tools in order to detect hepatic abnormalities in children with obesity in a timely manner, since the few available long-term studies have shown that the remission of SH in children and adolescents who participated in an intervention program was 30% in the case in which they did not receive any supplementation or medication; in contrast, with supplementation, remission was 58% and, with medication, it was 41% [49]. Given that we know that progressive damage to the hepatic parenchyma characterizes these alterations, and that the regression of the damage may not be complete, we feel the need to carry out an intentional and early screening of this comorbidity in pediatric patients with obesity to reinforce intervention strategies. It is also imperative to join efforts to try to describe pathophysiological mechanisms that will help us understand the phenomenon and propose potential target molecules for the development of specific therapies.

Some limitations must be considered in the present study. First, the sample size was small. However, it was sufficient for identifying metabolic phenotypes that are consistent with the findings of other studies and the pathophysiology of MASLD, even though these metabolites are not yet routinely used. On the other hand, a liver biopsy was not performed, which we know is the gold standard for diagnosing steatosis and hepatic fibrosis; nonetheless, FibroScan^®^ has been proposed as a non-invasive, painless, reliable, and reproducible method for diagnosing liver damage in children and adolescents [15]. Stiffness, which is determined using transient elastography and considered a consequence of the fibrotic process, is closely related to the histological biopsy findings of fibrosis in children [16,27,50]. Even though we could not evaluate the presence of SH due to a lack of liver biopsies, SH has been described as a progression stage in MASLD that may or may not be associated with fibrosis. Interestingly, previous study conducted mainly on Latin children living in the USA revealed that all children with SH also had fibrosis [8]. Another limitation is that there may have been a selection bias since several participants were referred to the Pediatric Obesity Clinic and Wellness Unit because of obesity-related comorbidities. Considering the mentioned limitations, our results seem to be indicative of a biology system’s pathological approach, and more studies are certainly needed to evaluate whether these metabotypes can be used as reliable biomarkers in other settings and populations. Furthermore, it would be interesting to evaluate, using longitudinal intervention studies, whether the metabolomic signatures found in this study are modified as liver abnormalities improve or whether they persist when liver damage regression is not observed.

A strength of this study is that all patients belonged to Class 1 obesity (which provides internal validity), they had similar genetic–environmental backgrounds, and they presented similar durations with respect to obesity. Moreover, we evaluated glucose metabolism using the Matsuda index, which more accurately reflects hepatic and muscle glucose sensitivity compared to those that consider a single blood determination. Additionally, to our knowledge, this is the first study aimed at identifying a metabotype for MASLD stages in Mexican children living with obesity.

## 5. Conclusions

According to our findings, MASLD phenotype changes as this dysfunction progresses, involving a switch in amino acid use. The assessment of ALT, proline, alanine, and Matsuda Index could be considered as metabolic signatures of MASLD in children living with obesity. However, more studies are necessary to broaden the knowledge about metabolic alterations in MASLD in order to prevent and identify this pathology during childhood, as well as its comorbidities, in addition to improving clinical treatment.

## Figures and Tables

**Table 1:** Demographic, clinical, biochemical, and metabolomic characteristics of children enrolled in the study.

Variable	Non-MASLD *n* = 14 Mean (SD)	MASLD *n* = 44 Mean (SD)	MASLD + Fibrosis *n* = 16 Mean (SD)	*p*-Value
Age (years)	10.46 ± 1.39	11.31 ± 2.39	11.69 ± 2.24	0.32
Duration of obesity (years)	4.73 ± 4.28	5.57 ± 4.12	4.15 ± 2.60	0.49
Weight (kg)	51.80 ± 10.42	61.58 ± 14.08	62.54 ± 13.22	0.053
Height (cm)	142.69 ± 7.54	148.55 ± 10.43	150.11 ± 11.55	0.12
BMI z-Score	1.88 ± 0.17	2.05 ± 0.29	2.07 ± 0.41	0.17
Waist circumference (cm)	80.63 ± 9.37 ^a^	87.48 ± 8.26 ^b^	88.85 ± 8.59 ^b^	0.023
Lean body mass (kg)	36.16 ± 4.78	41.17 ± 7.39	41.71 ± 8.43	0.72
Body Fat Mass (kg)	15.03 ± 6.27	19.82 ± 7.29	20.45 ± 5.54	0.061
Systolic BP (mmHg)	103.54 ± 9.30	105.56 ± 9.78	108.30 ± 14.32	0.49
Diastolic BP (mmHg)	66.67 ± 8.36	69.40 ± 7.21	69.25 ± 11.25	0.60
Glucose (mg/dL)	89.30 ± 5.43	89.44 ± 8.55	94.00 ± 6.07	0.17
Uric acid (mg/dL)	5.03 ± 1.21	5.58 ± 0.96	5.92 ± 0.98	0.36
Total cholesterol (mg/dL)	158.61 ± 28.32	158.82 ± 24.86	158.87 ± 26.16	0.46
Triglycerides (mg/dL)	141.07 ± 61.20	168.68 ± 86.37	149.68 ± 108.30	0.72
HDL-Chol (mg/dL)	39.07 ± 3.88	38.88 ± 8.42	37.75 ± 7.10	0.38
LDL-Chol (mg/dL)	102.30 ± 22.98	100.95 ± 17.89	105.75 ± 22.43	0.28
ALT IU/L	18.38 ± 4.94 ^a^	29.02 ± 19.02 ^b^	36.81 ± 25.57 ^b^	0.014
AST IU/L	23.84 ± 3.78	26.88 ± 8.73	31.00 ± 13.11	0.22
GGT IU/L	16.15 ± 5.36	19.28 ± 8.67	20.06 ± 9.69	0.69
Insulin IU/L	16.53 ± 11.11	18.34 ± 9.6	21.46 ± 8.23	0.29
HOMA-IR	3.63 ± 2.49	4.20 ± 2.27	5.00 ± 1.99	0.23
Matsuda-ISI	3.48 ± 2.53	3.23 ± 2.22	2.07 ± 1.07	0.051
CAP dB/m	176.60 ± 45.03 ^a^	279.69 ± 37.90 ^b^	287.06 ± 56.17 ^b^	<0.001
kPa	3.97 ± 0.59 ^a^	3.91 ± 0.72 ^a^	6.25 ± 1.80 ^b^	<0.001
Glycine	100.81 ± 22.85	100.39 ± 16.90	103.83 ± 16.90	0.31
Arginine	29.23 ± 4.85	29.01 ± 4.00	31.89 ± 6.59	0.10
Citrulline	9.23 ± 1.72	9.39 ± 1.46	9.41 ± 2.29	0.45
Proline	63.12 ± 8.69 ^a^	67.56 ± 16.18 ^a^	81.29 ± 21.18 ^b^	0.005
Alanine	115.77 ± 23.38 ^a^	119.43 ± 18.86 ^a^	137.54 ± 20.19 ^b^	0.014
ASA	1.75 ± 0.14	1.73 ± 0.20	1.81 ± 0.22	0.39
Tiglylcarnitine (C5:1)	0.03385 ± 0.004	0.03333 ± 0.0054	0.03375 ± 0.005	0.28
Pubertal Tanner stage	*n* (%)	*n* (%)	*n* (%)	*p*-value
Prepubertal	8 (57.1)	17 (38.6)	4 (25.0)	0.526
Early puberty	3 (21.4)	15 (34.1)	6 (37.5)	
Advanced puberty	3 (21.4)	12 (27.3)	6 (37.5)	

The *p*-values were estimated with the normalized concentrations of metabolites and biochemical parameters. Abbreviations: SD: standard deviation; ASA: arginosuccinate. ^a^ and ^b^ denote significant mean differences between groups. Groups with the same letter are not significantly different from each other; groups with different letters are statistically distinct.

**Table 2:** Evaluated logistic regression models to identify the phenotypes of MASLD.

Model Tested	ROC Area
**MASLD phenotype**	
Model 1: ASA + AC5:1 + glycine + arginine + citrulline	0.64
Model 2: Model 1 + ALT	0.68
Model 3: AC5:1 + glycine + arginine + citrulline + ALT	0.68
Model 4: AC5:1 + glycine + arginine + ALT	0.69
Model 5: glycine + arginine + ALT	0.69
**MASLD + fibrosis phenotype**	
Model 1: ALT + proline + alanine	0.73
Model 2: ALT + proline + alanine + Matsuda Index	0.80
Model 3: ALT + proline + alanine + Matsuda Index + AST	0.82

## Data Availability

The data presented in this study are available upon request from the corresponding author.
